# Association of cognitive function with increased risk of cancer death and all-cause mortality: Longitudinal analysis, systematic review, and meta-analysis of prospective observational studies

**DOI:** 10.1371/journal.pone.0261826

**Published:** 2022-01-07

**Authors:** Somayeh Rostamian, Saskia le. Cessie, Koen A. Marijt, J. Wouter Jukema, Simon P. Mooijaart, Mark A. van Buchem, Thorbald van Hall, Jacobijn Gussekloo, Stella Trompet

**Affiliations:** 1 Department of Radiology, Leiden University Medical Centre, Leiden, The Netherlands; 2 Department of Gerontology and Geriatrics, Leiden University Medical Centre, Leiden, The Netherlands; 3 Department of Medicine, National Heart & Lung Institute, Imperial College London, London, United Kingdom; 4 Department of Clinical Epidemiology, Leiden University Medical Centre, Leiden, The Netherlands; 5 Department of Clinical Oncology, Leiden University Medical Centre, Leiden, The Netherlands; 6 Department of Cardiology, Leiden University Medical Centre, Leiden, The Netherlands; Seoul National University, REPUBLIC OF KOREA

## Abstract

**Background:**

Disturbed cognitive function is associated with several causes of mortality; however, the association between cognitive function and the risk of cancer death has not been extensively investigated yet. We aimed to evaluate the association of cognitive function with the risk of cancer death and all-cause mortality in the PROspective Study of Pravastatin in the Elderly at Risk (PROSPER) and Leiden 85-plus Study. Additionally, a systematic review and meta-analysis of longitudinal studies were conducted to evaluate the association of cognitive function and risk of cancer death.

**Methods:**

Risk of cancer death and all-cause mortality were reported using hazard ratios (HRs) with 95% confidence interval (CI) in tertiles of cognitive function of PROSPER and Leiden85-Plus Study. Additionally, PubMed, Embase, Web of Science, Cochrane, PsycINFO, Academic Search Premier, CINHAL, and Emcare were searched up to November 1^st^, 2020 to perform a systematic review and meta-analysis. The relative risks (RRs) with 95%CI of cancer death per each standard deviation lower performance in cognitive measurements were calculated.

**Results:**

Participants of PROSPER had 1.65-fold (95%CI 1.11–2.47) greater risk of cancer death (P for trend = 0.016) and 1.85-fold (95%CI 1.46–2.34) higher risk of all-cause mortality (P for trend<0.001), in multivariable models. Results of the Leiden-85 Plus Study showed that subjects with MMSE score below 24 had a lower chance of cancer death (HR 0.79, 95%CI 0.36–1.70, P for trend = 0.820) but had 2.18-fold (95%CI 1.57–3.02) higher risk of all-cause mortality compared to the reference group (P for trend<0.001). Besides, the results of systematic review and meta-analysis showed that per each standard deviation lower performance in cognitive function, individuals were at a 10% higher chance of cancer death (RR 1.10, 95%CI 1.00–1.20, P-value = 0.044).

**Conclusions:**

Lower cognitive function performance is associated with a marginally increased risk of cancer death, in line with a significantly greater risk of all-cause mortality.

## Introduction

Research indicated that older individuals with covert or overt vascular injuries might be at increased risk of cancer development and mortality because of shared risk factors and common pathogenesis [1]. A growing body of evidence suggests that cancer death and cardiovascular mortality have shared risk factors, including smoking, excess body weight, poor diet, diabetes, hypertension, and hyperlipidemia [2–6]. A distinct number of studies have presented that adherence to cancer prevention guidelines for controlling tobacco smoking, body weight, diet, physical activity, and alcohol consumption is associated with a significantly lower risk of cancer death as well as cardiovascular and all-cause mortality [7, 8].

Next to that, longitudinal studies have stated that impaired cognitive function and different types of dementia are associated with higher risk of cardiovascular and all-cause mortality in older subjects, independent of confounders including socioeconomic status, history of vascular disease, and cardiovascular risk factors [9–12]. Accordingly, we previously evaluated the association of various cognitive domains with the incidence of cardiovascular events as well as all-cause and cause-specific mortality in different older populations [13–15].

Although the link between lower cognitive function with cardiovascular and all-cause mortality has been studied previously [16–18], only a few longitudinal studies have investigated whether cognitive function measured before cancer diagnosis might also be associated with the risk of death due to cancer, particularly among middle-aged and older individuals [17–21]. However, the populations of interest, methodological approaches, findings, and subsequent interpretation of outcomes were not consistent across all the studies.

In this study, we aimed to evaluate the association of cognitive function with the risk of cancer death and all-cause mortality in the Prospective Study of Pravastatin in the Elderly at Risk (PROSPER) and the Leiden 85-plus Study. Additionally, we conducted a systematic review and meta-analysis of published studies combined with these two studies to obtain the precise and constant magnitude of the association between cognitive function and death risk due to cancer.

## Methods

### PROSPER study

#### Study population

From December 1997 to May 1999, 5,804 older adults (men and women aged 70–82 years) at cardiovascular risk from Scotland, Ireland, and the Netherlands were invited to participate in the Prospective Study of Pravastatin in the Elderly at Risk (PROSPER). The institutional ethics review boards of all centres approved the protocol, and all participants gave written informed consent. The protocol was consistent with the Declaration of Helsinki. The study design and inclusion procedure of the PROSPER have been described in detail elsewhere [22, 23]. Briefly, PROSPER was a prospective randomized, double-blind placebo-controlled trial, which intended to evaluate whether pravastatin treatment decreases the risk of cardiovascular events in older individuals with either pre-existing vascular diseases (coronary, cerebral, or peripheral) or at increased risk of such diseases due to hypertension, diabetes mellitus or smoking. Participants with impaired cognitive function (Mini-Mental State Examination (MMSE) score of less than 24 out of 30) were excluded from the study at the time of enrolment. Moreover, subjects who were diagnosed with any cancer, five years before the initiation of the trial, were excluded from the study. In this study, we included 5,683 individuals with at least one cognitive test result at baseline.

#### Cognitive function assessment

Four cognitive tests were applied to evaluate four major domains of cognitive function. To measure selective attention, the Stroop Colour-Word test was applied in three stages. First, participants were asked to read or name black printed patches. At the next stage, subjects were asked to name coloured patches synchronized with the name of colours. At the last stage, participants should name the colour of the patches, which were printed in different colours from the name of the patches. To measure processing speed, a paper-based Letter Digit Substitution Test was applied. In this test, subjects were asked to write digits near letters based on the pattern provided at the top of the test sheet in 60 seconds. Lastly, immediate and delayed memory domains were tested by applying Picture Learning Test. Fifteen pictures were presented three times, two seconds each. Participants had to recall as many as possible, and the average of the recalled picture was reported as immediate. After 20 minutes, participants had to recall the pictures that indicated delayed memory. Scores of each test were converted to Z-score to standardize the results of cognitive tests, and for each participant, a composite cognitive score was created by averaging the Z-scores of four tests. The composite cognitive score was applied as a continuous variable in the PROSPER and the results were reported in tertiles (low, middle, and high) of the composite cognitive scores.

#### Outcomes

All endpoints of the PROSPER trial were assessed by the Endpoints Committee and were blinded to study medication. The main endpoint of the original PROSPER study was a combination of coronary and cerebrovascular events. Cancer death was assessed by Endpoint Committee in the PROSPER as a tertiary outcome. Dates of death were obtained from the Dutch municipal registry, and specific causes of death were obtained from the Central Bureau of Statistics of The Netherlands. All endpoints were adjudicated by the independent local clinical events committee of PROSPER. The underlying cause of death from a death certificate was coded according to the International Classification of Diseases and Related Disorders, 10^th^ revision, 1992. In this study, we considered cancer death and all-cause mortality as our main outcomes. The mean follow-up was 3.2 years.

#### Covariates

Age, sex, educational attainment, and country of origin as demographic and socioeconomic characteristics of participants along with smoking habits were recorded. Alcohol intake was measured as units of drink per week in the past month (one unit is equal to 60 millilitres distilled spirits, 170 millilitres wine, or 300 millilitres beer). Systolic and diastolic blood pressures were measured using sphygmomanometers, and the use of hypertensive medication was recorded at baseline. Fasting venous blood samples for biochemical and hematologic factors checks were collected. Body mass index was calculated as weight (in kilograms) divided by standing height (in meters squared). Apolipoprotein phenotypes were determined in plasma samples by Western blotting. The concentration of total serum cholesterol (millimole per litter) was measured at baseline in blood. History of diabetes mellitus, which was either known or identified by fasting blood glucose more than seven millimoles per litter, was recorded. History of all vascular diseases (coronary, cerebral, peripheral) was documented at the time of recruitment.

#### Statistical analyses

Baseline characteristics of subjects were reported as mean with standard deviation (SD) or median with interquartile range (if the data was skewed) for continuous variables and frequency with a percentage for categorical variables in the total population. The risk for cancer death and all-cause mortality per tertile of cognitive function was estimated with Cox regression and reported by hazard ratio (HR) with 95% confidence interval (CI) in three models. In the first step, results were reported without adjustment (crude model). In the second model, analyses were adjusted for age, sex, educational attainment, and country of origin (adjusted model 1). In the last step, analyses were further adjusted for current smoking, alcohol intake, body mass index, presence of apolipoprotein E4 phenotype, total serum cholesterol, systolic blood pressure, history of antihypertensive medication, history of diabetes mellitus, and history of vascular diseases (adjusted model 2). P for trend was calculated as a continuous variable in the regression model. All analyses were conducted using SPSS statistical software (SPSS for Windows, version 20, SPSS Inc., Chicago, IL).

### Leiden 85-plus study

#### Study population

Leiden 85-plus Study was a prospective population-based study of inhabitants of Leiden district, the Netherlands. The study design and characteristics of the cohort have previously been described in detail [24, 25]. In brief, between September 1^st^, 1997, and September 1^st^, 1999, 705 people from the 1912–1914 birth cohorts living in the city of Leiden who reached the age of 85 years were eligible to participate. There were no selection criteria based on health and demographic characteristics, and no exclusion criteria were used. Oral and written information about the study was provided, and informed consent was obtained from all subjects. Overall, 92 individuals refused to contribute, and 14 died before enrolment, and therefore, 599 subjects (response rate of 85%) participated in the study. Within a month after their 85^th^ birthday, a physician or a research nurse contacted individuals by phone to request their participation. The medical ethical committee of the Leiden University Medical Centre approved the study.

#### Cognitive function assessment

The MMSE test, which is a commonly used cognitive test as a diagnostic tool for detecting cognitive impairment and dementia, according to the definition in the Diagnostic and Statistical Manual of Mental Disorders [26], was administered to all participants [27]. The MMSE is a paper‐based test with a maximum score of 30. The lower scores indicate higher cognitive disturbance [26]. In this study, the results were reported in three groups of the MMSE based on clinical cut-off points: MMSE score equal to or above 27 points was considered as preserved cognitive function; MMSE score between 26 to 24 was indicative of mild cognitive impairment and resulted equal to or below 23 was indicated severe cognitive impairment.

#### Outcomes

In this study, we measured cancer death and all-cause mortality as our outcomes. Subjects were followed for cancer death and all-cause mortality for five years after the time of recruitment. Cause of death was classified as the International Classification of Diseases and Related Disorders, 10^th^ revision, 1992, and the date of death was found in the civic registries, and the cause of the death was obtained from CBS (the Netherlands Centraal Bureau voor de Statistiek).

#### Covariates

Demographic status data and the medical history of all participants were recorded at baseline.

Education was dichotomized as either low education and unskilled profession or high education and skilled profession. Body mass index and blood pressure were measured by trained personnel. All participants were interviewed for the history of smoking. Diabetes mellitus was considered present when included in the records of the primary care physician, when non-fasting glucose concentrations were more than 11.0 millimoles per litter, or when a participant was using anti-diabetic medication according to their pharmacy records. Hypertension was defined as the history of hypertension, or the use of antihypertensive medication at baseline. History of cardiovascular diseases, including atrial fibrillation, myocardial infarction, and cerebral accidents was obtained from general practitioners or nursing home physicians at baseline.

#### Statistical analyses

Baseline characteristics of subjects were reported as mean with SD for continuous variables and frequency with the percentage for categorical variables. The risk for cancer death and all-cause mortality were calculated by Cox regression and reported by HR with 95%CI in three stages. Firstly, we calculated hazard ratios for the association between cognitive function and outcomes (crude model). In the second model, analyses were adjusted for sex and education (adjusted model 1). Finally, analyses were further adjusted for current smoking, body mass index, total cholesterol, systolic blood pressure, history of antihypertensive medication, history of diabetes mellitus, history of atrial fibrillation, and history of cardiovascular diseases (adjusted model 2). P for trend was calculated as a continuous variable in the regression model. All analyses were conducted using SPSS statistical software (SPSS for Windows, version 20, SPSS Inc., Chicago, IL).

### Systematic review and meta-analysis

We used the preferred reporting items for systematic reviews and meta-analyses (PRISMA) statement as a guideline for this study [28].

#### Literature search strategy

We electronically searched several databases, including PubMed, Embase, Web of Science, Cochrane, PsycINFO, Academic Search Premier, CINHAL, and Emcare, up to November 1^st^, 2020, to include cohort studies considering the association between cognitive performance prior cancer mortality in adult populations. Search terms provided in S1 File (contains all the supporting tables).

#### Eligibility criteria and study selection

Inclusion and exclusion criteria are presented in S1 File (contains all the supporting tables). We set no search restrictions for follow-up time and study size. Additionally, we applied no language restriction on our search strategy; however, in the end, we only included English language cohort studies. Two members of the study team independently evaluated all titles and abstracts to identify eligible studies (S.R. and K.A.M). We obtained the full text if at least one of the reviewers arbitrated a study to be appropriated to our selective criteria. We reviewed reference lists of included studies to identify other relative studies. Disagreements on inclusion were resolved by consensus.

#### Data extraction

The following information was extracted from the included articles: author name, year of publication, number of participants, follow-up time, number of death due to cancer, overall mortality, sexual set percentage, age at the baseline of the study, exclusion of dementia at the baseline and cognitive tests. In each eligible study, data on the association between cognitive function and cancer death was estimated with hazard ratios and confidence intervals.

#### Quality assessment

The methodological quality of included studies was assessed by using previously explained criteria [29, 30]. The items of the tool assessed study quality within the domains of the study population, study attrition, data collection, and data analysis (S1 File, contains all the supporting tables). In total, 11 criteria were considered, and each quality criterion was rated as zero or one. Therefore, the maximum possible score was 11.

#### Data analysis

To make effect estimates comparable between studies, we reported the effect as relative risk (RR) per SD lower performance in cognitive scores for all included studies. We used the method reported by Viechtbauer [31] to calculate the estimation of RR per SD of the underlying normal distribution, assuming a log-linear relation on the underlying normal distribution scale between the cognitive test and the risk of death due to cancer. To apply the method, the numbers of cases per category were needed, which were given in the articles. After the calculation of RR for each study, a meta-analysis was performed using a random-effects model. The random-effects model was applied because it takes into account the variability between studies. All statistical analyses were performed using R version 3.6.2. We calculated the quantity I^2^ to describe the degree of heterogeneity [32].

## Results

### PROSPER study

Table 1 shows the baseline demographic status and clinical characteristics of participants. In this study, we included 5,683 individuals with at least one cognitive test result at baseline. Among those, 5,056 (89.0%) participants had completed profile of cognitive tests. The mean age was 75.3 years, and 2,937 (51.7%) of them were female.

**Table 1:** Characteristics of PROSPER participants.

Characteristics	Values (n = 5,683)
Age, years, mean (SD)	75.32 (3.35)
Female, n (%)	2937 (51.68)
Age left school, mean (SD)	15.14 (2.04)
Current smoking, n (%)	1533 (26.98)
Alcohol intake, u/m, median (IQR)	1 (0–7)
Body mass index, kg/m2, mean (SD)	26.85 (4.20)
Total cholesterol, mmol/L, mean (SD)	5.68 (0.91)
Systolic blood pressure, mmHg, mean (SD)	154.61 (21.89)
Diastolic blood pressure, mmHg, mean (SD)	83.74 (11.45)
APOE4, n (%)	1243 (21.87)
Antihypertensive treatment, n (%)	3518 (61.90)
History of diabetes mellitus, n (%)	611 (10.75)
History of vascular diseases, n (%)	2504 (44.06)

**Abbreviations:** n: number; SD: Standard Deviation; u/m: unite per month; IQR: Interquartile Range; kg/m2: kilogram-meter squared; mmHg: millimeters of mercury; mmol/L: millimoles per liter; APOE e4: apolipoprotein E4 (APOE4).

Table 2 shows the number and percentage of cancer death and all-cause mortality dependent on the level of composite cognitive score. During the follow-up time, 202 (3.6%) cases of cancer death and 589 (10.4%) cases of all-cause mortality were reported. In the fully adjusted analysis of the total population, subjects had 27% (HR 1.27, 95%CI 1.04–1.54) higher chance of death due to cancer (P = 0.018) and 35% (HR 1.35, 95%CI 1.22–1.52) all-cause mortality (P<0.001) per one SD lower performance in the continuous composite cognitive score. When tertiles of performance in cognitive function came to consideration, individuals in the middle tertile of the composite cognitive score had around 46% (HR 1.46, 95%CI 0.98–2.18) higher risk, and participants in the lowest tertile of the composite cognitive score had about 65% (HR 1.65, 95%CI 1.11–2.47) greater risk of cancer death (P for trend = 0.016). Moreover, our results showed that individuals in the middle tertile of the composite cognitive score had about 52% (HR 1.52, 95%CI 1.19–1.93) elevated risk, and participants in the lowest tertile of the composite cognitive score had around about 85% (HR 1.85, 95%CI 1.46–2.34) increased risk of all-cause mortality (P for trend<0.001).

**Table 2:** Risk of cancer death and all-cause mortality in relation to tertiles of composite cognitive score of PROSPER.

	Continuous composite cognitive score (n = 5,683)		Tertiles of composite cognitive score (n = 5,683)			
	Continuous	*P-value*	Highest tertile (n = 1894)	Middle tertile (n = 1895)	Lowest tertile (n = 1894)	*P for trend*
***Cancer Death***						
Number (%)	202 (3.55)	*-*	47 (2.48)	66 (3.48)	89 (4.70)	*-*
Crude model, HR (95%CI)	1.37 (1.15–1.61)	*<0*.*001*	1.00 (ref.)	1.43 (0.98–2.08)	2.01 (1.41–2.86)	*<0*.*001*
Model 1, HR (95%CI)	1.33 (1.12–1.61)	*0*.*001*	1.00 (ref.)	1.40 (0.93–1.98)	1.91 (1.32–2.75)	*<0*.*001*
Model 2, HR (95%CI)	1.27 (1.04–1.54)	*0*.*018*	1.00 (ref.)	1.46 (0.98–2.18)	1.65 (1.11–2.47)	*0*.*016*
***All-cause Mortality***						
Number (%)	589 (10.36)	*-*	121 (6.39)	194 (10.24)	274 (14.47)	*-*
Crude model, HR (95%CI)	1.56 (1.43–1.72)	*<0*.*001*	1:00 (ref.)	1.64 (1.31–2.06)	2.42 (1.94–2.99)	*<0*.*001*
Model 1, HR (95%CI)	1.45 (1.32–1.61)	*<0*.*001*	1:00 (ref.)	1.50 (1.19–1.89)	2.06 (1.65–2.57)	*<0*.*001*
Model 2, HR (95%CI)	1.35 (1.22–1.52)	*<0*.*001*	1:00 (ref.)	1.52 (1.19–1.93)	1.85 (1.46–2.34)	*<0*.*001*

**Abbreviations:** n: number; HR: Hazard Ratio; CI: Confidence Interval

**Model 1:** adjusted for age, sex, education and country. **Model 2:** model 1 further adjusted for body mass index, smoking, alcohol intake, total cholesterol, apolipoprotein E4, systolic blood pressure, antihypertensive treatment, statin treatment, history of diabetes mellitus, history of vascular diseases.

### Leiden 85-plus study

Table 3 shows the baseline demographic status and clinical characteristics of participants. All participants were 85 years old, and 396 (66.1%) subjects were female.

**Table 3:** Characteristics of study population of Leiden 85-plus study.

Characteristics	Values (n = 599)
Male, n (%)	396 (66.11)
Low education or unskilled profession, n (%)	386 (64.44)
Current Smoking, n (%)	96 (16.03)
Body mass index, kg/m2, mean (SD)	27.20 (4.47)
Total cholesterol, mmol/L, mean (SD)	5.71 (1.13)
Systolic blood pressure, mmHg, mean (SD)	155.13 (18.66)
Diastolic blood pressure, mmHg, mean (SD)	76.70 (9.53)
Antihypertensive treatment, n (%)	260 (43.41)
History of diabetes mellitus, n (%)	92 (15.36)
Arterial fibrillation, n (%)	56 (9.35)
History of cardiovascular events, n (%)	402 (67.11)

**Abbreviations:** n: number; kg/m2: kilogram per meter squared; SD: Standard Deviation; mmol/L: millimoles per litter; mmHg: millimetres of mercury.

Table 4 shows the number and percentage of cancer death and all-cause mortality dependent on the level of MMSE score. During the follow-up time, 55 (9.8%) cases of cancer death and 281 (50.3%) cases of all-cause mortality were reported. In the multivariable model of the total population, no association between performance with MMSE scores and cancer death was found (HR 0.99, 95%CI 0.92–1.05; P = 0.650). In contradiction, each point lower score of MMSE performance was associated with higher risk of all-cause mortality (HR 1.09, 95%CI 1.06–1.11; P<0.001). When we considered clinical cut-off points of MMSE, participants in the middle-performance group had around 49% (HR 0.51, 95%CI 0.23–1.13) lower risk, and participants with the lowest performance in the MMSE test had about 21% (HR 0.79, 95%CI 0.36–1.70) lesser risk of cancer death (P for trend = 0.820). Individuals with middle performance in MMSE had around 23% (HR 1.23, 95%CI 0.87–1.74) increased risk, and participants with the lowest performance in the MMSE test had more than two-fold (HR 2.18, 95%CI 1.57–3.02) greater risk of all-cause mortality (P for trend<0.001).

**Table 4:** Risk of cancer death and all-cause mortality depending on MMSE scores of Leiden 85-plus study.

	Continuous MMSE score (n = 599)		Group of MMSE scores based on clinical cut-off points (n = 599)			
	Continuous (n = 559)	*P-value*	MMSE ≥27 (n = 265)	MMSE: 24–26 (n = 146)	MMSE ≤23 (n = 184)	*P for trend*
***Cancer Death***						
Number (%)	55 (9.84)	*-*	32 (11.90)	9 (6.16)	14 (7.61)	*-*
Crude model, HR (95%CI)	1.01 (0.91–1.06)	*0*.*810*	1.00 (ref.)	0.54 (0.26–1.13)	0.85 (0.46–1.59)	*0*.*419*
Model 1, HR (95%CI)	0.98 (0.93–1.04)	*0*.*537*	1.00 (ref.)	0.59 (0.27–1.25)	1.07 (0.55–2.09)	*0*.*895*
Model 2, HR (95%CI)	0.99 (0.92–1.05)	*0*.*650*	1.00 (ref.)	0.51 (0.23–1.13)	0.79 (0.36–1.70)	*0*.*820*
***All-cause Mortality***						
Number (%)	281 (50.26)	*-*	96 (35.69)	63 (43.15)	122 (66.30)	*-*
Crude model, HR (95%CI)	1.08 (1.06–1.10)	*<0*.*001*	1:00 (ref.)	1.28 (0.93–1.76)	2.53 (1.93–3.31)	*<0*.*001*
Model 1, HR (95%CI)	1.09 (1.06–1.10)	*<0*.*001*	1:00 (ref.)	1.30 (0.94–1.79)	2.60 (1.94–3.47)	*<0*.*001*
Model 2, HR (95%CI)	1.09 (1.06–1.11)	*<0*.*001*	1:00 (ref.)	1.23 (0.87–1.74)	2.18 (1.57–3.02)	*<0*.*001*

**Abbreviations:** n: number; HR: Hazard Ratio; CI: Confidence Interval

**Model 1:** adjusted for sex and education. **Model 2:** model 1 further adjusted for body mass index, current smoking, total cholesterol, systolic blood pressure, arterial fibrillation, antihypertensive treatment, history of diabetes mellitus and history of cardiovascular diseases.

### Systematic review and meta-analysis

#### Literature search

**Fig 1:**
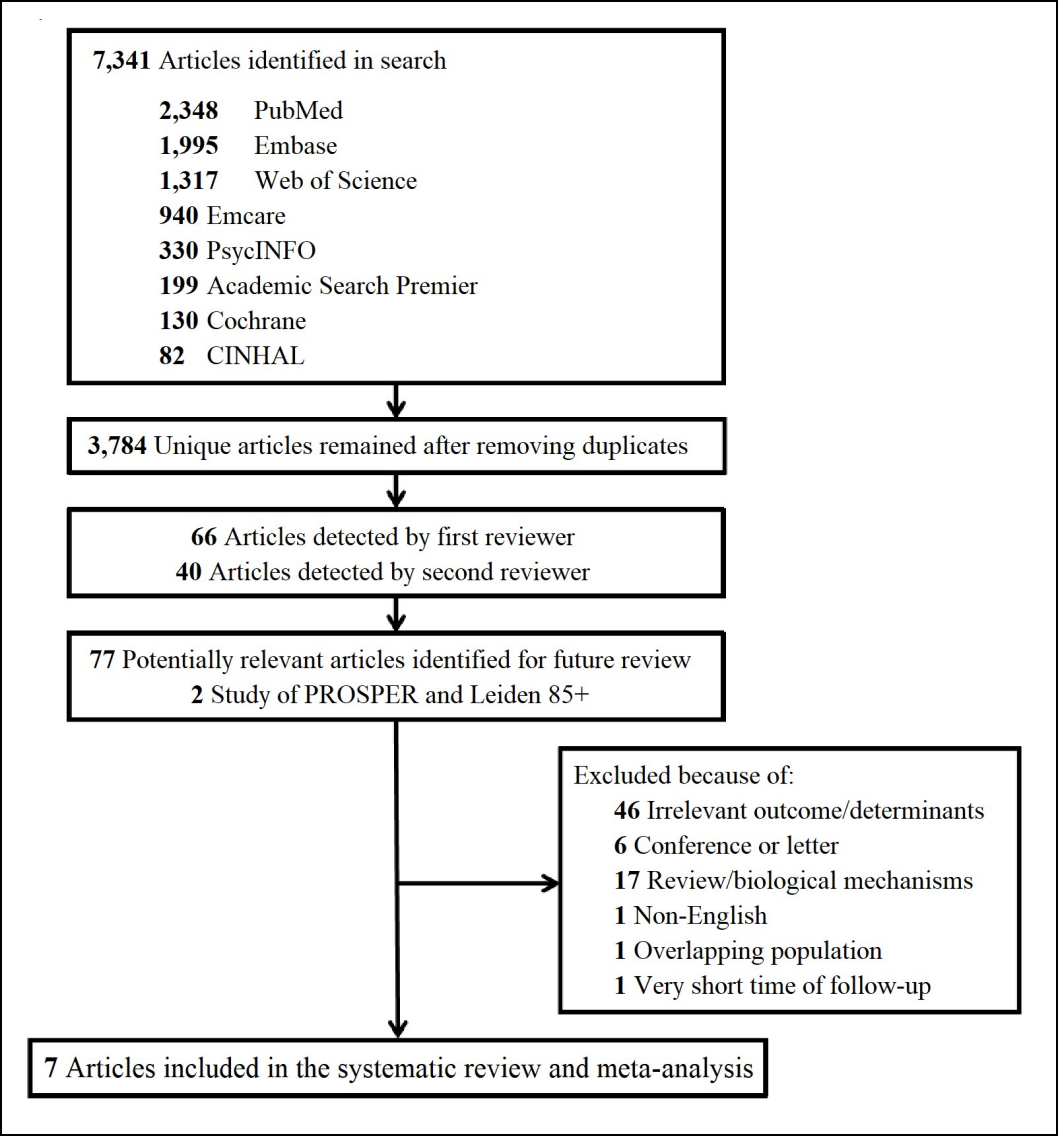
The literature search for the systematic review.

The literature search yielded in various databases resulted in 7,341 studies in which 3,784 of them were unique. After performing title and abstract screening, studies were identified by the first (S.R.) and second (K.A.M.) reviewers. After removing duplicates and full-text screening, five articles met the entry criteria, and results of the PROSPER and Leiden 85-plus Study were considered to complete the systematic review. A flow diagram of the search strategy is provided in Fig 1.

#### Study characteristics

The characteristics of the included studies are presented in Table 5. In total, 1,545 cancer deaths were reported among 25,493 participants from seven longitudinal studies. Follow-up times ranged from 3.2 to 25 years. Only two studies (Katsoulis et al. [20] and PROSPER) had follow-up time shorter than five years. Three studies excluded subjects with dementia at the time of recruitment (Perna et al. [17], Yaffe et al. [21], and PROSPER). Only one study included middle age and older people together (Betty et al. [19]), and all others considered individuals over 60 years old or older. Two studies used a composite score of several cognitive tests (Betty et al. [19] and PROSPER), and the rest used a single cognitive measurement. Among those five studies, one study applied cognitive telephone screening instruments (Perna et al. [17]), and the four remaining studies used MMSE to assess cognitive performance.

**Table 5:** Characteristics of studies included in systematic review and meta-analysis.

Author/Year	Country	Follow-up time (Year)	Number of participants	Number of cancer death (%)	Number of all-cause mortality (%)	Female sex (%)	Age at baseline	Baseline dementia excluded	Cognitive tests	Quality Assessment (out of 11)
Katsoulis et al. 2014 [20]	Greece	4	733	17 (2.3)	86 (11.1)	63.8	60.0+	NO	MMSE	7
Takata et al. 2014 [18]	Japan	10	205	17 (8.3)	120 (58.5)	57.1	85.0	NO	MMSE	7
Batty et al. 2014 [19]	England	9	9,204	509 (5.5)	1,488 (16.2)	54.1	50–100	NO	WLT, WFT, LCT	9
Perna et al. 2015 [17]	Germany	6.1	1,622	82 (5.1)	231 (14.2)	59.6	73.9 (SD 2.8)	YES	COGTEL	11
Yaffe et al. 2016 [21]	United States	25	7,447	663 (8.9)	4,451 (59.8)	100	65.0+	YES	MMSE	11
PROSPER 2020	Scotland, Ireland, and the Netherlands	3.2	5,683	202 (3.6)	589 (10.4)	51.7	75.3 (SD 3.4)	YES	Stroop, Letter-Digit, IPWLT, DPWLT	10
Leiden 85+ 2020	The Netherlands	5	599	55 (9.2)	281 (50.3)	66.1	85.0	NO	MMSE	10

**Abbreviations:** SD: Standard Deviation; MMSE: Mini-Mental State Examination; WLT: Word-List Learning Test; WFT: Word Finding Task; LCT: Letter Cancellation Test; COGTEL: Cognitive Telephone Screening Instrument; IPWLT: Immediate Picture-Word Learning Test; DPWLT: Delayed Picture-Word Learning Test.

From a total of 11 points, the overall quality assessment scores ranged from seven to eleven points, and the average was about nine, which indicates that included studies have high quality (Table 5).

#### Study results

**Fig 2:**
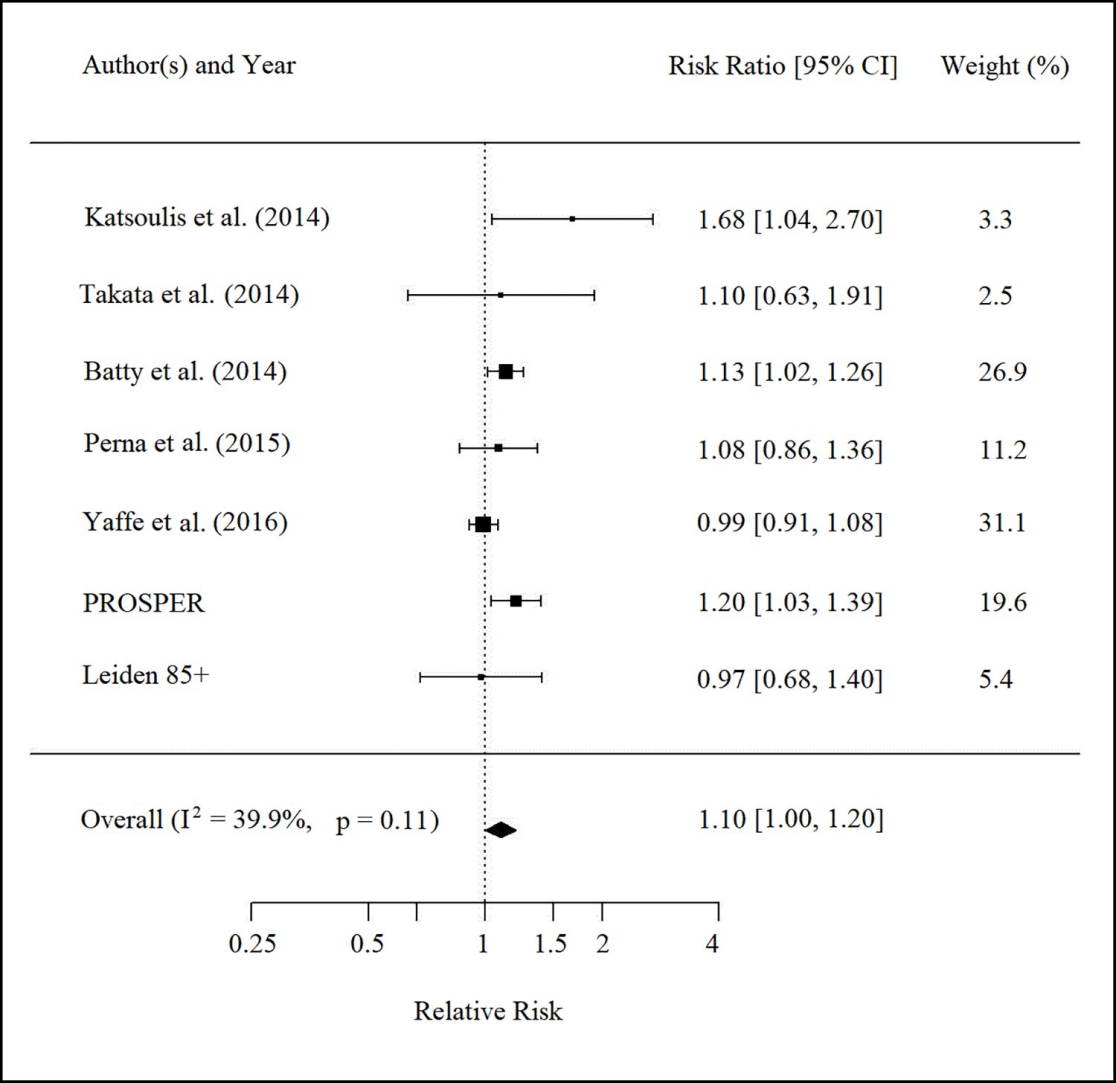
The relative risk of cancer death per standard deviation lower performance in cognitive tests.

Findings from studies are provided in Fig 2. Results of 25,493 subjects of seven studies pooled, and the risk of cancer death was evaluated per each SD lower performance in cognitive function. Three studies showed a significant association between lower performance in cognitive function and increased mortality risk due to cancer (Katsoulis et al. [20], Betty et al. [19], and PROSPER). After pooling the estimates from individual studies, the results indicated that each SD lower performance in a cognitive test was poorly associated with a 10 percent higher risk of cancer death (RR 1.10, 95%CI 1.00–1.20; P-value 0.044); however, heterogeneity testing revealed moderate heterogeneity (P for heterogeneity = 0.11; I^2^ = 39.9%). The funnel plot was asymmetrical, which confirmed a possibility for publication bias.

## Discussion

In this study, we show that participants of the Prospective Study of Pravastatin in the Elderly at Risk (PROSPER) who had either pre-existing vascular diseases or were at increased risk of such diseases had higher risk of all-cause mortality and cancer death and if they were at the lowest composite cognitive score compared to those who were at the highest tertile. Consequently, lower performance in MMSE tests was associated with the higher risk of all-cause mortality in the participants of the Leiden 85-plus Study, but there was no association between performance in cognitive function and cancer death.

To our knowledge, this is the first systematic review and meta-analysis to investigate the association between cognitive performance and risk of cancer death. Combined with the findings of the PROSPER and Leiden 85-plus Study, we observed a marginal relation in lower performance of cognitive function with a greater risk of cancer death.

Our findings are in line with other research revealing the importance of cognitive performance and several causes of mortality in older subjects [9–12]. During decades of studies, much attention has been given to the association between dementia and other health outcomes. However, investigations during recent years show that poor cognitive function, even in the absence of dementia, is independently associated with the different causes of mortality [14, 33, 34]. An inverse association between cognitive function with the risk of all-cause and cause-specific mortality has been indicated earlier from various studies. Research has shown that impairment in different domains of cognitive function is associated with overall mortality, including vascular and non-vascular causes [14–16]. The consistency of the findings suggests that cognitive dysfunction associated with various causes of mortality is a reflection of the underlying diseases [35]. Though we showed that lower performance in cognitive function associates with cancer death in the PROSPER, we failed to present this association in the Leiden 85-plus population. Since the prevalence of mortality in the Leiden 85-plus Study population is high due to cardiovascular and non-cardiovascular causes [14], the risk of mortality attributable to cancer is precluded by death due to other causes. Therefore, in the competing risks setting, the event of interest might be hampered by a competing event or censoring [36]. Moreover, the Leiden 85-plus Study included all subjects who reached the age of 85 years. There were no selection criteria based on health and demographic characteristics, and no exclusion criteria were used for the initiation of the study. However, in the PROSPER, there was an exclusion criterion that subjects should not be diagnosed with cancer five years prior to the initiation of the trial. This difference between the inclusion and exclusion criteria of these studies might be interfering with the health outcomes and could be considered as an explanation of the failure of presenting the association between cognitive function and cancer mortality in the Leiden 85-plus Study.

Cancer and vascular injuries establish an inordinate burden on public health and share several crucial modifiable risk factors, including tobacco smoking, obesity, poor lifestyle choices, diabetes, hypertension, and hyperlipidemia [2–6]. Common mechanisms underlying cardiovascular diseases and cancer elucidate the role of vascular health on the development of them [37].

On the contrary, in more recent longitudinal studies, the association of cognitive dysfunction and dementia with a higher risk of cardiovascular diseases and mortality has been extensively investigated. Thus, evaluation of cognitive function can be considered as a risk assessment for cardiovascular events and mortality in older subjects [9–12]. Therefore, an impaired cognitive function might be a sign of disruption of neural plasticity and cerebral integrity [38] as well as an early manifestation of covert vascular pathologies, putting subjects at a greater risk of cardiovascular events and mortality [13–15].

The association and mechanisms underlying cognitive function with the increased risk of cancer death and all-cause mortality have not been fully understood yet. Therefore, seeking possible explanations that could explicate this association is crucial. Behavioral and modifiable risk factors, including tobacco smoking, obesity, lack of physical activity, diabetes, heavy alcohol consumption, hypertension, and hyperlipidemia, are the first and foremost possible mechanisms underlying those conditions [2–6]. Besides, living in a low socioeconomic status community and unhealthy life choices are associated with greater cognitive impairment and a higher risk of death due to cancer and all-cause mortality [39–41]. Health literacy is the other prominent possible mechanism underlying cognitive function and cancer death [42, 43]. Health literacy defines as the ability and capacity of an individual for finding health information and medical services, communicating needs and preferences, understanding the choices and framework of information and services, and making a relevant decision to choose the best options based on the needs. People with poor health literacy are less likely to seek preventive strategies before the onset of diseases and have less ability to recognize the symptom of the disease, and have less tendency to stick to the treatment procedure [44, 45]. The other possible explanation for the observed association is poor adherence to medication and treatment due to cognitive deficits, which leads to decline in cognitive function as well as increases the risk of cancer death [46, 47]. Finally, system integrity might elucidate this association in which suggests that higher cognitive performance could be considered for a better constitution. That means the better cognitive function is a reflection of well-wired physiology, leading to better response to environmental stimuli [48].

This study has several strengths and limitations. As a strength, firstly, we reported data from two original studies and combined the results with findings of previously published research. Secondly, we included a relatively large number of participants from Europe, the United States, and South-Eastern Asia, the regions where aging and age-related disorders, such as cognitive dysfunction and cancer, are the leading public health concerns. As a limitation, the measurement of cognitive function was different for each study, which brings difficulties to compare findings. Another limitation of this systematic review and meta-analysis was the probability of publication bias due to the absence of unpublished data.

In conclusion, our results state that lower performance in cognitive function is associated with a higher risk of cancer death in the PROSPER participants and all-cause mortality in both PROSPER and Leiden 85-plus study population. Outcomes from our meta-analysis suggest that impaired cognitive performance is marginally associated with a higher risk of cancer death. These findings and previous research demonstrate that poor cognitive function in old age is a risk indicator for various causes of mortality, of which cancer death is one of them, as shown in the present study.

## Supporting information

S1 Checklist PRISMA 2009 checklist. (DOCX)

S1 File (DOCX)

## References

[1] van Kruijsdijk RC, van der Graaf Y, Peeters PH, Visseren FL, Second Manifestations of Adsg. Cancer risk in patients with manifest vascular disease: effects of smoking, obesity, and metabolic syndrome. Cancer Epidemiol Biomarkers Prev. 2013;22(7):1267–77. Epub 2013/05/17. doi: 10.1158/1055-9965.EPI-13-0090 . 23677576

[2] Barengo NC, Antikainen R, Harald K, Jousilahti P. Smoking and cancer, cardiovascular and total mortality among older adults: The Finrisk Study. Prev Med Rep. 2019;14:100875. Epub 2019/05/08. doi: 10.1016/j.pmedr.2019.100875 ; PubMed Central PMCID: PMC6488533. 31061784 PMC6488533

[3] Blaes A, Prizment A, Koene RJ, Konety S. Cardio-oncology Related to Heart Failure: Common Risk Factors Between Cancer and Cardiovascular Disease. Heart Fail Clin. 2017;13(2):367–80. Epub 2017/03/11. doi: 10.1016/j.hfc.2016.12.006 ; PubMed Central PMCID: PMC5547738. 28279422 PMC5547738

[4] Grossman E, Messerli FH, Boyko V, Goldbourt U. Is there an association between hypertension and cancer mortality? Am J Med. 2002;112(6):479–86. Epub 2002/04/18. doi: 10.1016/s0002-9343(02)01049-5 . 11959059

[5] Kopelman PG. Obesity as a medical problem. Nature. 2000;404(6778):635–43. Epub 2000/04/15. doi: 10.1038/35007508 . 10766250

[6] van Kruijsdijk RC, van der Wall E, Visseren FL. Obesity and cancer: the role of dysfunctional adipose tissue. Cancer Epidemiol Biomarkers Prev. 2009;18(10):2569–78. Epub 2009/09/17. doi: 10.1158/1055-9965.EPI-09-0372 . 19755644

[7] Kabat GC, Matthews CE, Kamensky V, Hollenbeck AR, Rohan TE. Adherence to cancer prevention guidelines and cancer incidence, cancer mortality, and total mortality: a prospective cohort study. Am J Clin Nutr. 2015;101(3):558–69. Epub 2015/03/04. doi: 10.3945/ajcn.114.094854 ; PubMed Central PMCID: PMC4340061. 25733641 PMC4340061

[8] McCullough ML, Patel AV, Kushi LH, Patel R, Willett WC, Doyle C, et al. Following cancer prevention guidelines reduces risk of cancer, cardiovascular disease, and all-cause mortality. Cancer Epidemiol Biomarkers Prev. 2011;20(6):1089–97. Epub 2011/04/07. doi: 10.1158/1055-9965.EPI-10-1173 . 21467238

[9] Newman AB, Sachs MC, Arnold AM, Fried LP, Kronmal R, Cushman M, et al. Total and cause-specific mortality in the cardiovascular health study. The journals of gerontology Series A, Biological sciences and medical sciences. 2009;64(12):1251–61. doi: 10.1093/gerona/glp127 ; PubMed Central PMCID: PMC2773812. 19723772 PMC2773812

[10] Gale CR, Martyn CN, Cooper C. Cognitive impairment and mortality in a cohort of elderly people. Bmj. 1996;312(7031):608–11. doi: 10.1136/bmj.312.7031.608 ; PubMed Central PMCID: PMC2350374. 8595334 PMC2350374

[11] Villarejo A, Benito-Leon J, Trincado R, Posada IJ, Puertas-Martin V, Boix R, et al. Dementia-associated mortality at thirteen years in the NEDICES Cohort Study. Journal of Alzheimer’s disease: JAD. 2011;26(3):543–51. doi: 10.3233/JAD-2011-110443 . 21694455

[12] Williams MM, Xiong C, Morris JC, Galvin JE. Survival and mortality differences between dementia with Lewy bodies vs Alzheimer disease. Neurology. 2006;67(11):1935–41. doi: 10.1212/01.wnl.0000247041.63081.98 . 17159097

[13] Rostamian S, van Buchem MA, Westendorp RG, Jukema JW, Mooijaart SP, Sabayan B, et al. Executive function, but not memory, associates with incident coronary heart disease and stroke. Neurology. 2015;85(9):783–9. Epub 2015/08/08. doi: 10.1212/WNL.0000000000001895 . 26245926

[14] Rostamian S, van Buchem MA, Jukema JW, Gussekloo J, Poortvliet RKE, de Cren AJM, et al. Lower Performance in Orientation to Time and Place Associates with Greater Risk of Cardiovascular Events and Mortality in the Oldest Old: Leiden 85-Plus Study. Front Aging Neurosci. 2017;9:307. Epub 2017/10/13. doi: 10.3389/fnagi.2017.00307 ; PubMed Central PMCID: PMC5623724. 29021754 PMC5623724

[15] Rostamian S, de Haan S, van der Grond J, van Buchem MA, Ford I, Jukema JW, et al. Cognitive Function in Dementia-Free Subjects and Survival in Old Age: The PROSPER Study. Am J Med. 2019;132(12):1466–74 e4. Epub 2019/06/23. doi: 10.1016/j.amjmed.2019.06.001 . 31228412

[16] O’Donnell M, Teo K, Gao P, Anderson C, Sleight P, Dans A, et al. Cognitive impairment and risk of cardiovascular events and mortality. Eur Heart J. 2012;33(14):1777–86. Epub 2012/05/04. doi: 10.1093/eurheartj/ehs053 . 22551598

[17] Perna L, Wahl HW, Mons U, Saum KU, Holleczek B, Brenner H. Cognitive impairment, all-cause and cause-specific mortality among non-demented older adults. Age Ageing. 2015;44(3):445–51. Epub 2014/12/04. doi: 10.1093/ageing/afu188 . 25468013

[18] Takata Y, Ansai T, Soh I, Awano S, Nakamichi I, Akifusa S, et al. Cognitive function and 10 year mortality in an 85 year-old community-dwelling population. Clin Interv Aging. 2014;9:1691–9. Epub 2014/10/23. doi: 10.2147/CIA.S64107 ; PubMed Central PMCID: PMC4199981. 25336934 PMC4199981

[19] Batty GD, Deary IJ, Zaninotto P. Association of Cognitive Function With Cause-Specific Mortality in Middle and Older Age: Follow-up of Participants in the English Longitudinal Study of Ageing. American journal of epidemiology. 2016;183(3):183–90. doi: 10.1093/aje/kwv139 ; PubMed Central PMCID: PMC4724091. 26803665 PMC4724091

[20] Katsoulis M, Kyrozis A, Trichopoulou A, Bamia C, Trichopoulos D, Lagiou P. Cognitive impairment and cancer mortality: a biological or health care explanation? Cancer causes & control: CCC. 2014;25(11):1565–70. doi: 10.1007/s10552-014-0460-9 . 25146445

[21] Yaffe K, Peltz CB, Ewing SK, McCulloch CE, Cummings SR, Cauley JA, et al. Long-term Cognitive Trajectories and Mortality in Older Women. The journals of gerontology Series A, Biological sciences and medical sciences. 2016;71(8):1074–80. doi: 10.1093/gerona/glw003 ; PubMed Central PMCID: PMC4945886. 26843186 PMC4945886

[22] Shepherd J, Blauw GJ, Murphy MB, Cobbe SM, Bollen EL, Buckley BM, et al. The design of a prospective study of Pravastatin in the Elderly at Risk (PROSPER). PROSPER Study Group. PROspective Study of Pravastatin in the Elderly at Risk. The American journal of cardiology. 1999;84(10):1192–7. doi: 10.1016/s0002-9149(99)00533-0 . 10569329

[23] Shepherd J, Blauw GJ, Murphy MB, Bollen EL, Buckley BM, Cobbe SM, et al. Pravastatin in elderly individuals at risk of vascular disease (PROSPER): a randomised controlled trial. Lancet. 2002;360(9346):1623–30. Epub 2002/11/30. doi: 10.1016/s0140-6736(02)11600-x . 12457784

[24] der Wiel AB, van Exel E, de Craen AJ, Gussekloo J, Lagaay AM, Knook DL, et al. A high response is not essential to prevent selection bias: results from the Leiden 85-plus study. J Clin Epidemiol. 2002;55(11):1119–25. Epub 2003/01/01. doi: 10.1016/s0895-4356(02)00505-x . 12507676

[25] van Exel E, Gussekloo J, Houx P, de Craen AJ, Macfarlane PW, Bootsma-van der Wiel A, et al. Atherosclerosis and cognitive impairment are linked in the elderly. The Leiden 85-plus Study. Atherosclerosis. 2002;165(2):353–9. Epub 2002/11/06. doi: 10.1016/s0021-9150(02)00253-8 . 12417287

[26] Creavin ST, Wisniewski S, Noel-Storr AH, Trevelyan CM, Hampton T, Rayment D, et al. Mini-Mental State Examination (MMSE) for the detection of dementia in clinically unevaluated people aged 65 and over in community and primary care populations. Cochrane Database Syst Rev. 2016;(1):CD011145. Epub 2016/01/14. doi: 10.1002/14651858.CD011145.pub2 . 26760674 PMC8812342

[27] van Vliet P, Westendorp RG, van Heemst D, de Craen AJ, Oleksik AM. Cognitive decline precedes late-life longitudinal changes in vascular risk factors. J Neurol Neurosurg Psychiatry. 2010;81(9):1028–32. Epub 2010/06/15. doi: 10.1136/jnnp.2009.182519 . 20543187

[28] Moher D, Liberati A, Tetzlaff J, Altman DG, Group P. Preferred reporting items for systematic reviews and meta-analyses: the PRISMA statement. Bmj. 2009;339:b2535. doi: 10.1136/bmj.b2535 ; PubMed Central PMCID: PMC2714657. 19622551 PMC2714657

[29] Stroup DF, Berlin JA, Morton SC, Olkin I, Williamson GD, Rennie D, et al. Meta-analysis of observational studies in epidemiology: a proposal for reporting. Meta-analysis Of Observational Studies in Epidemiology (MOOSE) group. Jama. 2000;283(15):2008–12. doi: 10.1001/jama.283.15.2008 . 10789670

[30] Tooth L, Ware R, Bain C, Purdie DM, Dobson A. Quality of reporting of observational longitudinal research. American journal of epidemiology. 2005;161(3):280–8. doi: 10.1093/aje/kwi042 . 15671260

[31] Viechtbauer W. Conducting meta-analyses in R with the metafor package. Journal of Statistical Software. 2010;36 48(1548–7660). doi: 10.18637/jss.v036.i03

[32] Khoshdel A, Attia J, Carney SL. Basic concepts in meta-analysis: A primer for clinicians. International journal of clinical practice. 2006;60(10):1287–94. doi: 10.1111/j.1742-1241.2006.01078.x . 16981972

[33] Obisesan TO, Gillum RF. Cognitive function, social integration and mortality in a U.S. national cohort study of older adults. BMC geriatrics. 2009;9:33. doi: 10.1186/1471-2318-9-33 ; PubMed Central PMCID: PMC2724371. 19638207 PMC2724371

[34] Sachs GA, Carter R, Holtz LR, Smith F, Stump TE, Tu W, et al. Cognitive impairment: an independent predictor of excess mortality: a cohort study. Annals of internal medicine. 2011;155(5):300–8. doi: 10.7326/0003-4819-155-5-201109060-00007 . 21893623

[35] Lavery LL, Dodge HH, Snitz B, Ganguli M. Cognitive decline and mortality in a community-based cohort: the Monongahela Valley Independent Elders Survey. J Am Geriatr Soc. 2009;57(1):94–100. Epub 2008/11/20. doi: 10.1111/j.1532-5415.2008.02052.x ; PubMed Central PMCID: PMC2768614. 19016932 PMC2768614

[36] Lau B, Cole SR, Gange SJ. Competing risk regression models for epidemiologic data. Am J Epidemiol. 2009;170(2):244–56. Epub 2009/06/06. doi: 10.1093/aje/kwp107 ; PubMed Central PMCID: PMC2732996. 19494242 PMC2732996

[37] Koene RJ, Prizment AE, Blaes A, Konety SH. Shared Risk Factors in Cardiovascular Disease and Cancer. Circulation. 2016;133(11):1104–14. Epub 2016/03/16. doi: 10.1161/CIRCULATIONAHA.115.020406 ; PubMed Central PMCID: PMC4800750. 26976915 PMC4800750

[38] Jellinger KA, Attems J. Neuropathological approaches to cerebral aging and neuroplasticity. Dialogues Clin Neurosci. 2013;15(1):29–43. Epub 2013/04/12. doi: 10.31887/DCNS.2013.15.1/kjellinger ; PubMed Central PMCID: PMC3622466. 23576887 PMC3622466

[39] Wee LE, Yeo WX, Yang GR, Hannan N, Lim K, Chua C, et al. Individual and Area Level Socioeconomic Status and Its Association with Cognitive Function and Cognitive Impairment (Low MMSE) among Community-Dwelling Elderly in Singapore. Dementia and geriatric cognitive disorders extra. 2012;2(1):529–42. doi: 10.1159/000345036 ; PubMed Central PMCID: PMC3522450. 23277785 PMC3522450

[40] Saldana-Ruiz N, Clouston SA, Rubin MS, Colen CG, Link BG. Fundamental causes of colorectal cancer mortality in the United States: understanding the importance of socioeconomic status in creating inequality in mortality. American journal of public health. 2013;103(1):99–104. doi: 10.2105/AJPH.2012.300743 ; PubMed Central PMCID: PMC3518351. 23153135 PMC3518351

[41] Vineis P, Wild CP. Global cancer patterns: causes and prevention. Lancet. 2014;383(9916):549–57. doi: 10.1016/S0140-6736(13)62224-2 . 24351322

[42] Berkman ND, Sheridan SL, Donahue KE, Halpern DJ, Crotty K. Low health literacy and health outcomes: an updated systematic review. Annals of internal medicine. 2011;155(2):97–107. doi: 10.7326/0003-4819-155-2-201107190-00005 . 21768583

[43] Simmons RA, Cosgrove SC, Romney MC, Plumb JD, Brawer RO, Gonzalez ET, et al. Health Literacy: Cancer Prevention Strategies for Early Adults. Am J Prev Med. 2017;53(3S1):S73–S7. Epub 2017/08/19. doi: 10.1016/j.amepre.2017.03.016 . 28818249

[44] Federman AD, Sano M, Wolf MS, Siu AL, Halm EA. Health literacy and cognitive performance in older adults. Journal of the American Geriatrics Society. 2009;57(8):1475–80. doi: 10.1111/j.1532-5415.2009.02347.x ; PubMed Central PMCID: PMC2754116. 19515101 PMC2754116

[45] Dharmapuri S, Best D, Kind T, Silber TJ, Simpson P, D’Angelo L. Health literacy and medication adherence in adolescents. The Journal of pediatrics. 2015;166(2):378–82. doi: 10.1016/j.jpeds.2014.10.002 . 25454933

[46] Hayes TL, Larimer N, Adami A, Kaye JA. Medication adherence in healthy elders: small cognitive changes make a big difference. Journal of aging and health. 2009;21(4):567–80. doi: 10.1177/0898264309332836 ; PubMed Central PMCID: PMC2910516. 19339680 PMC2910516

[47] Hershman DL, Shao T, Kushi LH, Buono D, Tsai WY, Fehrenbacher L, et al. Early discontinuation and non-adherence to adjuvant hormonal therapy are associated with increased mortality in women with breast cancer. Breast cancer research and treatment. 2011;126(2):529–37. doi: 10.1007/s10549-010-1132-4 ; PubMed Central PMCID: PMC3462663. 20803066 PMC3462663

[48] Deary IJ. Looking for ’system integrity’ in cognitive epidemiology. Gerontology. 2012;58(6):545–53. doi: 10.1159/000341157 . 22907506

