# Supplementary material for: Association of cognitive function with increased risk of cancer death and all-cause mortality: Longitudinal analysis, systematic review, and meta-analysis of prospective observational studies
Source: PLoS One. 2022 Jan 7;17(1):e0261826. doi: 10.1371/journal.pone.0261826 (PMC8741047; doi:10.1371/journal.pone.0261826)
Supplement: S1 File — (DOCX) [file pone.0261826.s002.docx]

**Full Title: Association of cognitive function with increased risk of cancer death and all-cause mortality: Longitudinal analysis, systematic review, and meta-analysis of prospective observational studies**

**Short Title: Cognitive function associates with higher risk of cancer death and all-cause mortality**

Authors: Somayeh Rostamian, PhD ^1,2,3^; Saskia le. Cessie, PhD ^4^; Koen A. Marijt, PhD ^5^; J. Wouter Jukema, MD, PhD ^6^; Simon P. Mooijaart, MD, PhD ^2^; Mark A van Buchem, MD, PhD ^1^; Thorbald van Hall, PhD ^5^; Jacobijn Gussekloo, MD, PhD ^2^; Stella Trompet, PhD ^2^*.

Email of Authors: S.Rostamian@imperial.ac.uk; S.le_Cessie@lumc.nl; K.A.Marijt@lumc.nl; J.W.Jukema@lumc.nl; S.P.Mooijaart@lumc.nl; M.A.van_Buchem@lumc.nl; T.van_Hall@lumc.nl; J.Gussekloo@lumc.nl; [S.Trompet@lumc.nl](mailto:S.Trompet@lumc.nl).

^1^ Department of Radiology, Leiden University Medical Centre, Leiden, the Netherlands; ^2^ Department of Gerontology and Geriatrics, Leiden University Medical Centre, Leiden, the Netherlands; ^3^ National Heart & Lung Institute, Imperial College London, United Kingdom, ^4^ Department of Clinical Epidemiology, Leiden University Medical Centre, Leiden, the Netherlands; ^5^ Department of Clinical Oncology, Leiden University Medical Centre, Leiden, The Netherlands; ^6^ Department of Cardiology, Leiden University Medical Centre, Leiden, the Netherlands.

Corresponding author: Stella Trompet, PhD; Assistant Professor at Leiden University Medical Centre, Leiden, the Netherlands.

Mailing address: Albinusdreef 2, 2333 ZA, Leiden, The Netherlands

Tel: +31715261312 Email: [S.Trompet@lumc.nl](mailto:S.Trompet@lumc.nl)

**Search Terms**

| **Participants** | **cognition OR cognitive OR cognitive function OR cognitive defect OR cognitive dysfunction OR cognitive impairment OR mild cognitive impair OR dementia OR vascular dementia OR senile OR alzheimer’s disease OR primary progressive aphasia OR primary progressive nonfluent aphasia OR creutzfeldt-jakob OR frontotemporal lobar degeneration OR huntington OR kluver-bucy syndrome OR lewy body disease OR parkinson OR comprehension OR consciousness OR imagination OR dreams OR fantasy OR intuition OR neurocognitive disorders OR amnesia OR delirium** |
| --- | --- |
| **Investigations** | **cognitive test OR cognition test OR cognitive exam OR cognition exam OR mini mental state examination OR MMSE OR mental status schedule OR neuropsychological tests OR cognitive performance OR cognitive OR memory OR executive function OR executive control OR attention OR processing speed OR orientation OR cognitive decline OR cognitive trajectories** |
| **Outcomes** | **risk OR risk factors OR genetic predisposition to disease OR causality OR comorbidity OR incidence OR cause of death AND neoplasms OR cancer OR carcinoma OR neoplasm OR malignant OR tumour OR metastasis AND mortality** |

**Inclusion and Exclusion Criteria of Systematic Review and Meta-Analysis**

| **Inclusion Criteria**   1. Original and peer-reviewed clinical studies with adult participants 2. Literature published up to 1^st^ February 2019 3. Prospective studies 4. Cognitive function as the determinant of interest 5. Cancer death as the outcome of interest 6. Cognitive function assessed by neuropsychological tests 7. Mortality assessed by medical reports, and death certificates 8. Results were estimated by valid statistical analysis | **Exclusion Criteria**   1. Animal Studies 2. Reviews, conference abstracts, web pages, letters 3. Not prospective design 4. Determinant rather than cognitive function 5. Outcome rather that cancer death 6. Qualitative studies 7. Overlapping population |
| --- | --- |

**Quality assessment scores**

| **Reference** | **Hypothesis or Aim (specific for cognitive function and cancer death)** | | **Inclusion/ExclusionCriteria** | **Response rate higher than 75%** | **Information responders vs. non-responders** | **Participant characteristics** | **Follow-up at least for 5 years** |
| --- | --- | --- | --- | --- | --- | --- | --- |
| **Katsoulis et al.** | | 1 | 1 | 0 | 1 | 1 | 0 |
| **Takata et al.** | | 1 | 0 | 0 | 1 | 1 | 1 |
| **Batty et al.** | | 1 | 0 | 1 | 1 | 1 | 1 |
| **Perna et al.** | | 1 | 1 | 1 | 1 | 1 | 1 |
| **Yaffe et al.** | | 1 | 1 | 1 | 1 | 1 | 1 |
| **PROSPER** | | 1 | 1 | 1 | 1 | 1 | 0 |
| **Leiden 85+** | | 1 | 1 | 1 | 1 | 1 | 1 |

| **Reference** | **The validity of the cognitive test and/or**  **measurements** | | **Excluding subjects with dementia at the baseline** | **Cancer death reported by official statements** | **Statistical**  **methods** | **Correction for**  **potential**  **confounders** | **Overall quality**  **score** |
| --- | --- | --- | --- | --- | --- | --- | --- |
| **Katsoulis et al.** | | 1 | 0 | 0 | 1 | 1 | 7 |
| **Takata et al.** | | 1 | 0 | 1 | 1 | 0 | 7 |
| **Batty et al.** | | 1 | 0 | 1 | 1 | 1 | 9 |
| **Perna et al.** | | 1 | 1 | 1 | 1 | 1 | 11 |
| **Yaffe et al.** | | 1 | 1 | 1 | 1 | 1 | 11 |
| **PROSPER** | | 1 | 1 | 1 | 1 | 1 | 10 |
| **Leiden 85+** | | 1 | 0 | 1 | 1 | 1 | 10 |
